# Supplementary material for: Competition between crystal growth and intracrystalline chain diffusion determines the lamellar thickness in semicrystalline polymers
Source: Nat Commun. 2022 Jan 10;13:119. doi: 10.1038/s41467-021-27752-0 (PMC8748680; doi:10.1038/s41467-021-27752-0)
Supplement: Supplementary file 1 — Supplementary Information [file 41467_2021_27752_MOESM1_ESM.pdf]

Supplementary Information
Competition between crystal growth and intracrystalline chain diffusion determines the lamellar thickness in semicrystalline polymers

Martha Schulz, Mareen Schäfer, Kay Saalwächter,* and Thomas Thurn-Albrecht *

Institut für Physik, Martin-Luther-Universität Halle-Wittenberg, 06099 Halle, Germany

E-mail: kay.saalwaechter@physik.uni-halle.de; thomas.thurn-albrecht@physik.uni-halle.de

Characterization of ICD in Polyoxymethylene by NMR

Full NMR results

Table 1 shows the values of the activation energies E_a and of $\log\tau_0$ (cf. eq. (2) in the main paper) of all samples in dependence of the crystallization temperatures T_c. The given error values correspond to uncertainties from the Arrhenius fits. Further systematic deviations due to temperature dependent widths of the τ_c-distributions are neglected. To give an impression on the variations of the correlation time $\langle\tau_\mathrm{c}\rangle$ between different samples, $\langle\tau_\mathrm{c}\rangle$ is given at a fixed temperature $T = 155\,°\mathrm{C}$, in the range of interest. The values of $\langle\tau_\mathrm{c}\rangle(T_\mathrm{c})$ of the samples POM130 and POM212 are listed in the last column. These values are displayed in Figure 2 and were used for the calculation of τ_stem.

Additionally, an injection molded sample purchased from Goodfellow, POM207 was investigated as received. This sample was used for the complementary rheological experiments. As for the other POM samples, the molecular weight of POM207 was determined using poly(methylmethacrylate) calibration and HFIP/ 0.05 M KTFAC as solvent: M_w=207 kg mol^{-1}, M_n=61.6 kg mol^{-1}.

Contribution from spin diffusion

To illustrate the negligible contribution from spin diffusion, Figure 1 shows an extended version of the Arrhenius plot shown in Figure 1c of the main paper. It includes τ_c values measured at much lower temperatures, where they illustrate an approach towards a temperature-independent plateau value attributable to spin diffusion. The low-temperature values are more than one order of magnitude larger than the longest τ_c of interest, and they extrapolate to a plateau contribution even two decades above the range of interest.

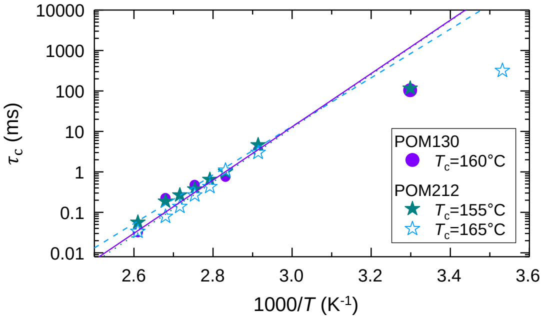

Figure 1: Extended version of the Arrhenius plot shown in Figure 1c. τ_c values measured at much lower temperatures are included.

Table 1: Activation parameters and correlation times of ICD of all POM samples. POM130 and POM212 were isothermally crystallized. POM207 was investigated as received.

Sample	E_a (kJ mol^{-1})	$\log(\tau_0/1s)$	$\langle\tau_c\rangle$ ($T = 155\,°\text{C}$) (ms)	$\langle\tau_c\rangle$ (T_c) (ms)
POM130				
$T_c = 155°\text{C}$	117 ± 4	-17.4 ± 0.6	0.81	0.81
$T_c = 160°\text{C}$	126 ± 16	-18.15 ± 1.85	0.52	0.35
$T_c = 165°\text{C}$	107 ± 6	-15.9 ± 0.9	1.31	0.66
$T_c = 168°\text{C}$	103 ± 3	-15.35 ± 0.35	1.74	0.74
average	113	-16.7	-	-
POM212				
$T_c = 155°\text{C}$	115 ± 7	-16.9 ± 1	1.32	1.32
$T_c = 160°\text{C}$	129 ± 6	-19.05 ± 0.95	0.61	0.40
$T_c = 165°\text{C}$	127 ± 5	-18.85 ± 0.65	0.47	0.21
$T_c = 168°\text{C}$	95 ± 10	-14.2 ± 0.6	2.48	1.13
average	117	-17.25	-	-
POM207	93	14.05 ± 0.75	1.86	-

Complementary rheological measurements

As many of the early investigations of the α_c-relaxation in POM were performed by dynamic mechanical analysis and as the reported values for the activation energy vary considerably, we decided to perfom some complementary rheological measurements in order to check the consistency of our NMR results with mechanical measurements. Since the preparation of samples suitable for rheology by compression moulding was difficult, we chose a commercially available injection moulded plate, POM207, from which a sample of suitable size was cut out. A rheometer ARES-G2 from TA Instruments in torsion geometry was used, sample dimensions were 30 mm x 10 mm x 1 mm. The experiments covered a range of $\omega = 0.1\,\text{rad}\,\text{s}^{-1}$ to $\omega = 50\,\text{rad}\,\text{s}^{-1}$ with a fixed strain of 0.1 % to ensure a linear deformation.

Figure 2a shows a temperature sweep of the loss modulus G'' measured with a frequency of 0.1Hz. Two relaxation peaks are visible. In the shown temperature range three relaxation peaks are known, often termed as α-, β- and γ-relaxation, where the amplitude of the β-process is very low[1]. We can therefore attribute the low temperature peak in Figure 2a to the γ-process and the high temperature peak to the α-process. A β-process is not clearly detectable. The α-peak is generally assigned to the intracrystalline chain dynamics and therefore called α_c-process. Frequency sweeps across this peak measured at different temperatures allow the construction of a master curve and the determination of the activation energy from the shift factors. As the direct determination of the shift factor from the mechanical data was ambiguous, we used a shift factor a_T based on an Arrhenius relation with the NMR value for the activation energy ($E_a = 93\,\text{kJ}\,\text{mol}^{-1}$). The master curve constructed in this way is shown in the upper part of Figure 2b and indicates that additional vertical shifting is necessary. The lower part of Figure 2b shows that a well-behaved master curve can be constructed by a manually adjusted additional vertical shift factor b_T. The vertical deviations are most likely a consequence of structural changes occuring during heating like e.g. surface melting or even partial melting of thinner lamellae. Nevertheless, the results show the consistency of mechanical measurements with NMR results and show at the same time, that NMR allows a much more reliable and precise determination of the activation energy and of the relaxation frequencies.

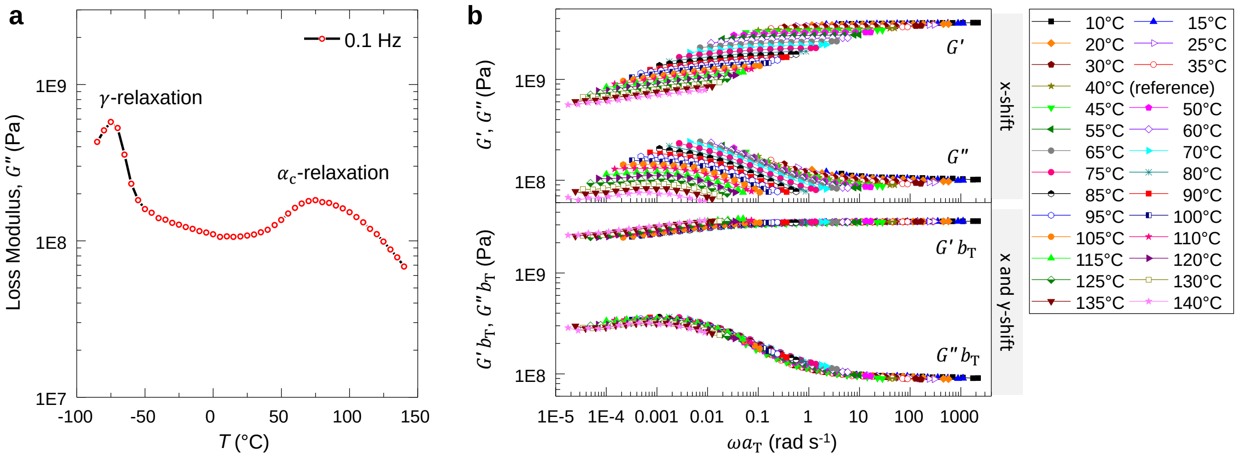

Figure 2: Rheological measurements for an injection molded plate of POM207. **a** Temperature sweep of the loss modulus G'' for a frequency $\omega/2\pi = 0.1\,\text{Hz}$ **b** Master curve of storage modulus G' and loss modulus G'' constructed from frequency sweeps measured at different temperatures using horizontal shifting (upper plot) and horizontal and vertical shifting (lower plot). The horizontal shift factor a_T was calculated using the NMR-value $E_\text{a} = 93\,\text{kJ}\,\text{mol}^{-1}$ and the vertical shift factor b_T was determined manually. The reference temperature $T_\text{ref} = 40\,°\text{C}$. The measurement series was started at the lower temperatures.

Calculation of the timescales τ_lc, $\langle\tau_\text{c}\rangle$ and τ_stem

The lamellar growth velocity μ of growing spherulites of PCL, POM and PEO, used for the calculation of τ_lc following equation (1) are given in Table 2. μ was measured by polarized light optical microscopy during isothermal crystallization at different T_c.

Table 3 shows the equations and the parameters used for the calculation of $\langle\tau_\text{c}\rangle$ and τ_stem for PCL, POM and PEO shown in Fig. 2 of the main paper. For POM the lamellar thickness at the end of the primary crystallization process was used to calculate τ_stem. For PEO τ_lc, $\langle\tau_\text{c}\rangle$ and τ_stem were already published in ref. (4). These d_c-values were recorded after crystallization over night. Further changes of d_c after the primary crystallization are negligible for the presentation in Figure 2 in the main paper.

Table 2: Crystallization velocity μ for samples of PCL, POM and PEO.

PCL66		PCL138		POM130		POM212		PEO53		PEO188	
T_c (°C)	μ (μm s^{-1})	T_c (°C)	μ (μm s^{-1})	T_c (°C)	μ (μm s^{-1})	T_c (°C)	μ (μm s^{-1})	T_c (°C)	μ (μm s^{-1})	T_c (°C)	μ (μm s^{-1})
34	0.363	34	0.247	156	6.287	156	3.307	44	13.897	45	18.969
36	0.288	36	0.179	158	2.799	158	1.572	46	12.075	46	14.378
38	0.199	38	0.120	160	1.493	160	0.684	48	8.881	47	12.692
40	0.129	40	0.084	162	0.894	162	0.210	50	6.117	48	10.496
42	0.078	42	0.044	164	0.316	164	0.059	52	4.324	49	8.575
44	0.049	44	0.024	166	0.101	165	0.032	54	1.337	50	6.548
46	0.033	46	0.013	168	0.013	166	0.020	56	0.776	51	5.119
48	0.014	48	0.005			168	0.005	58	0.176	52	0.176
50	0.009	50	0.002					60	0.020	53	0.020
										54	1.991
										55	1.403
										56	0.939
										57	0.586
										58	0.348
										59	0.189
										60	0.037

Table 3: Overview of equations and parameters used for the calculation of the timescales $\langle\tau_c\rangle$ and τ_{stem} for PCL, POM and PEO

<table>
<tr><td></td><td>PCL</td><td colspan="2">POM</td><td colspan="2">PEO</td></tr>
<tr><td>eq. $\langle\tau_c\rangle$</td><td rowspan="5">no hints for intra-crystalline chain dynamics by NMR; excluded up to 1s; source: ref. (2)</td><td colspan="2">$\langle\tau_c\rangle = \tau_0 \cdot \exp \frac{E_a}{k \cdot T}$</td><td colspan="2">$\langle\tau_c\rangle = \tau_0 \cdot \left(\frac{d_c}{4.6\text{nm}} + 0.77\right) \exp \frac{E_a}{RT}$</td></tr>
<tr><td>E_a and τ_0</td><td colspan="2">T_c- and M_w- dependent;
Table 1</td><td colspan="2">$E_a = 64.5\text{kJ mol}^{-1}$, $\tau_0 = 8 \cdot 10^{-18}$s
source: ref. (3)</td></tr>
<tr><td>eq. τ_{stem}</td><td colspan="4">$\tau_{stem} \approx \langle\tau_c\rangle \cdot {d_c}^2/\Delta {z_c}^2$</td></tr>
<tr><td>Δz_c (nm)</td><td colspan="2">$c/9 = 0.192$
(9_5 helix)</td><td colspan="2">$c/7 = 0.279$
(7_2 helix)</td></tr>
<tr><td>d_c (nm) (T_c (°C))</td><td>POM130
10.86(155)
11.34(160)
13.35(165)
17.04(168)</td><td>POM212
10.75(155)

14.29(168)</td><td>PEO53
21.12(45)
22.04(50)
25.19(55)
38.23(60)</td><td>PEO188
20.71(47.5)
27.35(52.5)
37.84(57.5)
61.19(61.5)</td></tr>
</table>

Structural analysis by SAXS - method and exemplary data

All structural data shown in the main paper (Porod parameter P, amorphous/crystalline thickness $d_{\mathrm{a/c}}$ with corresponding thickness distribution $\sigma_{\mathrm{a/c}}$ and long period $L = d_{\mathrm{a}} + d_{\mathrm{c}}$) were determined by a quantitative analysis of SAXS data as exemplary shown in Figure 3. The analysis method is described in detail in refs. (4, 5). Here, a brief account of the approach and important steps are given.

The small angle X-ray scattering signal of a semicrystalline polymer is related to the semicrystalline morphology.[6] Structure parameters like d_{c} and d_{a} can be determined directly from the interface distribution function (IDF), which can be calculated from the scattering signal by a cosine transform. For a one dimensional two-phase structure the SAXS intensity $I(s)$ of an isotropic sample is related to the IDF $K''(z)$ by[7]

$$K''(z) = 16\pi^3 \int_0^\infty \left[\lim_{s\to\infty} I(s)s^4 - I(s)s^4\right] \cos(2\pi s z)ds. \tag{1}$$

Here $s = (2\sin\Theta)/\lambda$ is the scattering vector. $\lim_{s\to\infty} I(s)s^{-4} = P$ is the so-called Porod constant.[8]

To calculate the IDF from real measured data affected by noise, the following procedure has been developed.[9] In a first step P is determined from the intensity curves using a fitting function of $I(s) = Ps^{-4} + c_1 s^2 + c_2$ for the range of large s as shown in the first column of Figure 3. Additionally to the Porod behavior (Ps^{-4}) a contribution by the amorphous halo ($c_1 s^2$) and a constant background caused by thermal density fluctuations of the amorphous phase have to be taken into account and subtracted. For the fit we take the condition $[K''(z=0) = 0]$ as an additional constraint.[10] With the value of P known, the IDF $K''(z)$ can be calculated from the measured data according to eq. 1, and d_{c}, d_{a} and L can be read off from the peak positions (compare to Fig. 3, column 3).

To obtain more complete structural information, we here used a recently introduced refined method of analysis based on quantitative modeling of the interface distribution function (IDF) by a series of distance distributions, representing the semicrystalline structure of the sample.

$$K''_{\mathrm{model}}(z) = \frac{O_{\mathrm{s}}\Delta\rho^2}{2}(h_{\mathrm{a}}(z) + h_{\mathrm{c}}(z) - 2h_{\mathrm{ac}}(z) + h_{\mathrm{aca}}(z) + ...) \tag{2}$$

Here $h_{\mathrm{a/c}}(z)$ are distributions of the amorphous and crystalline layer thickness, d_{a} and d_{c}. $h_{\mathrm{ac}}(z)$ is the thickness distribution of the long period $L = d_{\mathrm{a}} + d_{\mathrm{c}}$, and so on. To model the IDF $K''(z)$), we assume $h_{\mathrm{a/c}}(z)$ to be Gaussian distribution functions.

$$h_{\mathrm{a/c}}(z) = \frac{1}{\sqrt{2\pi}\sigma_{\mathrm{a/c}}} \exp -\frac{(z - d_{\mathrm{a/c}})^2}{2\sigma_{\mathrm{a/c}}^2} \tag{3}$$

Higher order distributions $h_{\mathrm{ac...}}(z)$ are convolutions of $h_{\mathrm{a}}(z)$ and $h_{\mathrm{c}}(z)$. O_{s} is the specific inner surface, $\Delta\rho$ denotes the electron density difference between crystalline and amorphous phases. Taking the inverse transform of eq. 1 an analytical model function for the integrand of eq. 1 can be calculated from a given $K''(z)$. The second column of Figure 3 shows exemplary data sets of the calculated integrand $16\pi^3(P - I(s)s^4)G$ together with the fitted model functions. To suppress the influence of noise at high scattering vectors, the data as well as the corresponding fit function were multiplied with a smoothing function $G(s) = \exp(-4\pi^2 s^2 \sigma^2)$. σ was kept constant and chosen as small as possible (PCL: 1.0 nm; POM: 0.8 nm; PEO: 1.4 nm). The corresponding IDFs $K''(z)$ calculated from the data as well as calculated from the fits are shown in the third column of Figure 3. With the described analysis we obtain in addition to the mean values of d_{a} and d_{c} the corresponding distributions widths $\sigma_{\mathrm{a/c}}$. Furthermore, the method also works for cases like the data shown for PCL, for which the distributions d_{c} and d_{a} overlap, which makes it difficult to directly read off the corresponding values from $K''(z)$ without decomposition into the individual components.

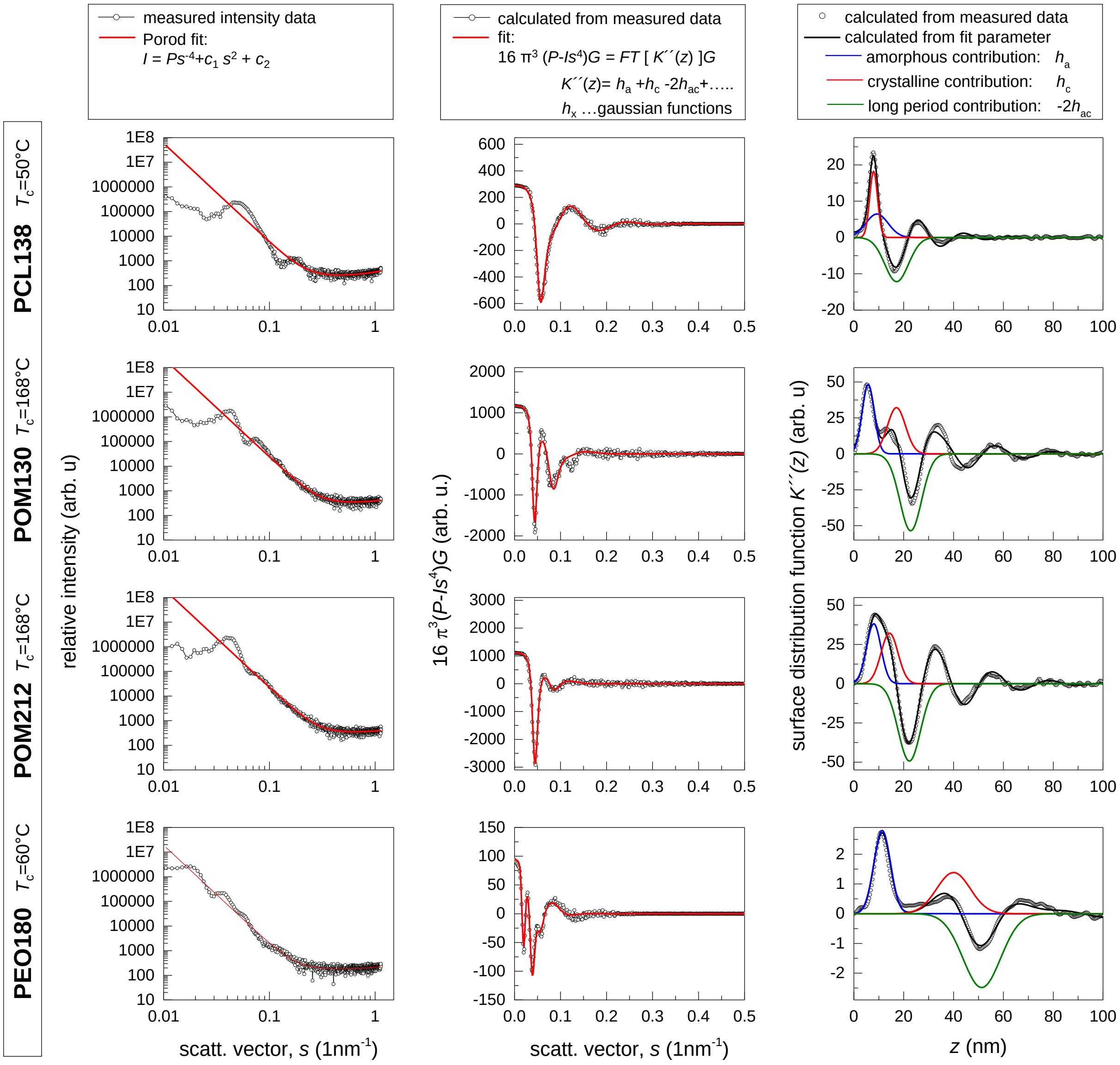

Figure 3: Quantitative analysis of SAXS data for PCL138, POM130, POM212 and PEO180. Exemplarily data sets are shown after isothermal crystallization with a supercooling of about 5 K. The measurements are taken at the end of the primary crystallization process. The first column shows the measured scattering curve and the corresponding Porod fit, following the Porod behavior (Ps^{-4}) and including intensity contributions by amorphous halo ($c_1 s^2$) and a constant contribution from thermal density fluctuations within the amorphous phase (c_2) The second column shows the function $16\pi^3(P - Is^4)G$ as calculated from column one. The fit is based on a model surface distribution function K'', described by a series of gaussian functions h_x, containing the characteristic structure parameters ($d_\mathrm{a}, \sigma_\mathrm{a}, d_\mathrm{c}, \sigma_\mathrm{c}$). The third column shows the resulting $K''(z)$ as calculated from the data and from the fit of column two. Knowing the values of $d_\mathrm{a}, \sigma_\mathrm{a}, d_\mathrm{c}$, and σ_c allows the decomposition of the IDF into the individual contributions as shown.

Additional presentations of the values for the crystal thickness

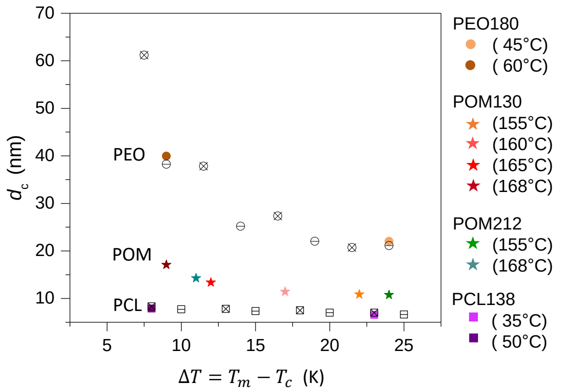

Figure 4: Lamellar thickness d_c at the end of primary crystallization vs. apparent supercooling ΔT. The figure shows the unscaled values of the crystal thickness used for the scaling plot in Figure 4b in the main paper. As above, the closed symbols are the data from Figure 4a in the main paper, open symbols represent already published data of PEO (open circles) and PCL (open squares) for two molecular weights (cross and line) measured at different T_cs.[4] The apparent supercooling was calculated with the DSC melting temperatures given in Table I in the main paper.

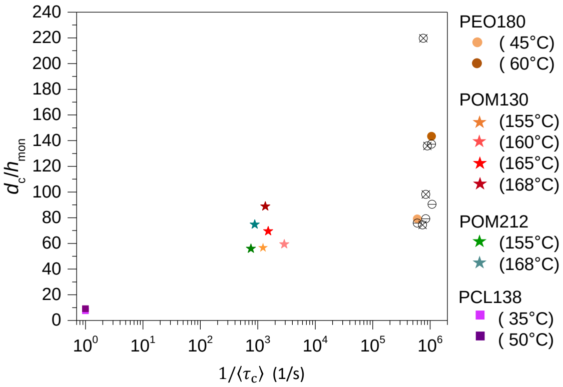

Figure 5: Normalized lamellar thickness d_c at the end of primary crystallization vs. the inverse timescale $1/\langle\tau_c\rangle$. The figure illustrates that scaling of the crystal thickness is not obtained in plot of the normalized crystal thickness vs. the inverse jump correlation time. Rather, the combined variable $\tau_{lc}/\langle\tau_c\rangle$ is necessary. The data are the same as in Fig. 4b of the main paper. For PCL a constant value of 1 s was taken for $\langle\tau_c\rangle$, corresponding to the lower limit of a possible jump correlation time.

References

(1) McCrum, N. G. Internal friction in polyoxymethylene. *Journal of Polymer Science* **54**, 561–568 (1961).

(2) Schäler, K., Achilles, A., Bärenwald, R., Hackel, C. & Saalwächter, K. Dynamics in crystallites of poly(ϵ-caprolactone) as investigated by solid-state NMR. *Macromolecules* **46**, 7818–7825 (2013).

(3) Kurz, R. *et al.* Intracrystalline jump motion in poly(ethylene oxide) lamellae of variable thickness: A comparison of NMR methods. *Macromolecules* **50**, 3890–3902 (2017).

(4) Schulz, M. *et al.* The underestimated effect of intracrystalline chain dynamics on the morphology and stability of semicrystalline polymers. *Macromolecules* **51**, 8377–8385 (2018).

(5) Seidlitz, A. & Thurn-Albrecht, T. *Polymer Morphology: Principles, Characterization, and Processing: Chapter 9. Small-Angle X-ray Scattering For Morphological Analysis Of Semicrystalline Polymers*, 151–164 (Wiley & Sons, New Jersey, 2016).

(6) Strobl, G. *The Physics Of Polymers. Concepts For Understanding Their Structures And Behavior* (Springer, Berlin, 2007), 3., rev. and expanded ed.

(7) Ruland, W. The evaluation of the small-angle scattering of lamellar two-phase systems by means of interface distribution functions. *Colloid and Polymer Science* **255**, 417–427 (1977).

(8) Porod, G. Die Röntgenkleinwinkelstreuung von dichtgepackten kolloiden Systemen. 1. Teil. *Kolloid-Zeitschrift and Zeitschrift fur Polymere* **124**, 83–114 (1951).

(9) Albrecht, T. & Strobl, G. Temperature-dependent crystalline-amorphous structures in linear polyethylene - surface melting and the thickness of the amorphous layers. *Macromolecules* **28**, 5827–5833 (1995).

(10) Albrecht, T. & Strobl, G. Temperature-dependent crystalline-amorphous structures in isotactic polypropylene - small-angle X-ray-scattering analysis of edge-bounded 2-phase systems. *Macromolecules* **28**, 5267–5273 (1995).
